# Termination of Sustained Ventricular Fibrillation During Radiofrequency Catheter Ablation

**DOI:** 10.1016/j.jaccas.2022.07.039

**Published:** 2022-09-21

**Authors:** Aalap Narichania, Pablo Salazar, Ryan Burris, Roderick Tung

**Affiliations:** University of Chicago Medicine, Center for Arrhythmia Care, Pritzker School of Medicine, Chicago, Illinois, USA

**Keywords:** catheter ablation, moderator band, premature ventricular complex, Purkinje, ventricular fibrillation, MCS, mechanical circulatory support, PMVT, polymorphic ventricular tachycardia, PVC, premature ventricular complexes, VF, ventricular fibrillation, VT, ventricular tachycardia

## Abstract

After a ST-segment elevation inferior myocardial infarction, a patient developed multiple drug-refractory ventricular fibrillation (VF), triggered by a stereotypic premature ventricular complex. During an episode of sustained VF, catheter ablation of the moderator band terminated VF, with transition into monomorphic ventricular tachycardia. To the best of our knowledge, this is the first-in-human report of termination of VF during delivery of radiofrequency energy, which suggests that the focal area on moderator band of Purkinje system had an active role in the perpetuation of VF. (**Level of Difficulty: Advanced.**)

## Central Illustration

**Figure:**
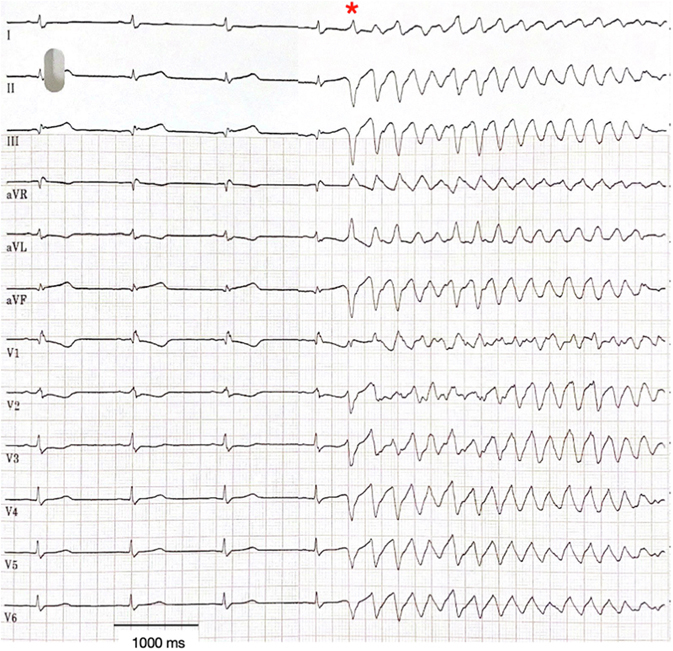

## History of Presentation

A 50-year-old man with a history of hypertension and diabetes presented to an outside hospital with an inferior ST-segment elevation myocardial infarction and underwent percutaneous coronary intervention to the right coronary artery. By report, he developed stuttering chest pain 1 month before presentation. Despite delayed revascularization with incomplete reperfusion, he developed polymorphic ventricular tachycardia (PMVT) and ventricular fibrillation (VF) requiring 3 separate episodes of resuscitation. Due to cardiogenic shock and right ventricular (RV) infarction, he was placed on mechanical circulatory support (MCS) and transferred to our institution.

**Learning Objectives**

• To be able to manage electrical storm after myocardial infarction.
• To understand that catheter ablation may have an important therapeutic role in drug refractory VF.
• To understand the purported mechanism of VF after myocardial infarction and examine the possibility of underlying Purkinje activity involved in both the initiation and maintenance of VF.

Shortly after arrival, he developed VF storm, which worsened his respiratory failure and biventricular heart failure. He was taken to the operating room, and MCS was upgraded to right-sided veno-venous extracorporeal membrane oxygenation (Protek Duo, LivaNova) and a higher-flow Impella 5.5 (Abiomed). He continued to have episodes of VF, which became incessant and refractory to intravenous amiodarone, lidocaine, and procainamide—despite over 10 defibrillation attempts within a 48-hour period.

On physical examination, the patient was in VF, was intubated, and mean arterial pressure was 50 to 60 mm Hg with MCS. Review of patient data was approved by the Institutional Review Board at the University of Chicago Medical Center.

## Differential Diagnosis

The differential diagnosis of the etiology of PMVT and VF includes myocardial ischemia, severe acidemia, torsades de pointes (PMVT in setting of genetic or acquired prolonged QT interval), catecholaminergic polymorphic ventricular tachycardia (VT), Brugada syndrome, and other channelopathies. In addition, short-coupled premature ventricular complexes (PVCs) can initiate VF in structurally normally hearts (idiopathic VF) or in a heart with cardiomyopathy from any cause including myocardial infarction (MI).

## Investigations

Chest radiograph revealed acute pulmonary edema. MCS devices were in appropriate anatomic positions. Coronary angiography revealed patent right coronary artery stents. The pH was 7.4 and lactate 1.0 despite refractory VF. Bedside echocardiography ruled out pericardial effusion, without evidence of mitral regurgitation or ventricular septal defect.

**Figure 1:**
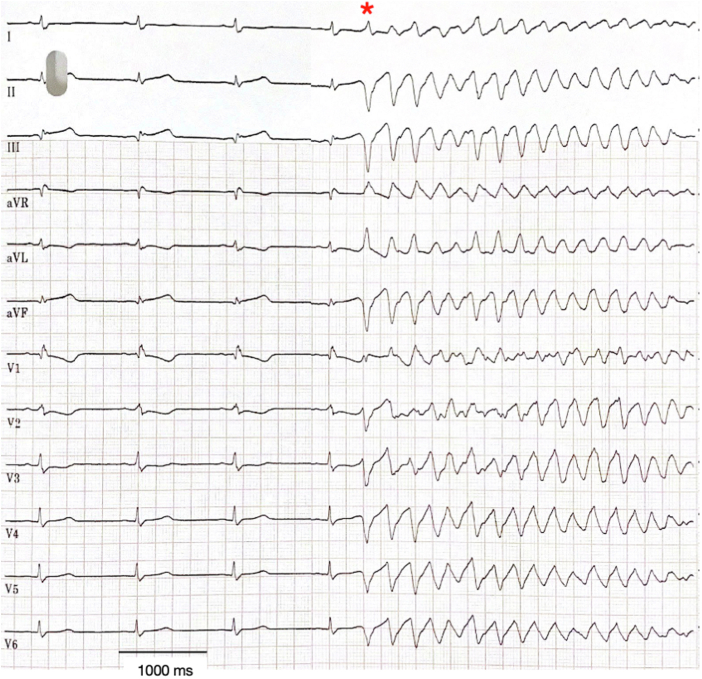
12-Lead Electrocardiogram of Spontaneous Ventricular Fibrillation Initiation 12-lead electrocardiogram shows sinus rhythm with a right bundle branch block. The QRS axis is normal. There are persistent ST-segment elevations in leads III and aVF with Q waves consistent with incomplete reperfusion. Midway through the tracing, ventricular fibrillation is initiated by a stereotypic premature ventricular complex **(asterisk)** with indeterminate V_1_ morphology with superior axis and dominant S waves, consistent with localization to the moderator band, septum, or distal left posterior fascicle toward the apex.

Review of telemetry and the ECG showed that VF was reproducibly initiated by a stereotypic PVC with indeterminate V_1_ morphology with superior axis and dominant S waves (Figure 1) consistent with localization to the moderator band, septum, or distal posterior fascicle. The QTc interval during sinus rhythm was 444 milliseconds.

## Management

Oral quinidine sulfate 324 mg via nasogastric tube was initiated. Defibrillation led to longer periods of sinus rhythm but VF recurred. The patient was not a candidate for durable left ventricular assist device or transplantation. Approximately 24 hours after VF storm at our institution, the patient was then brought to the electrophysiology laboratory for a compassionate ablation attempt to modify the PVC trigger initiating VF.

**Figure 2:**
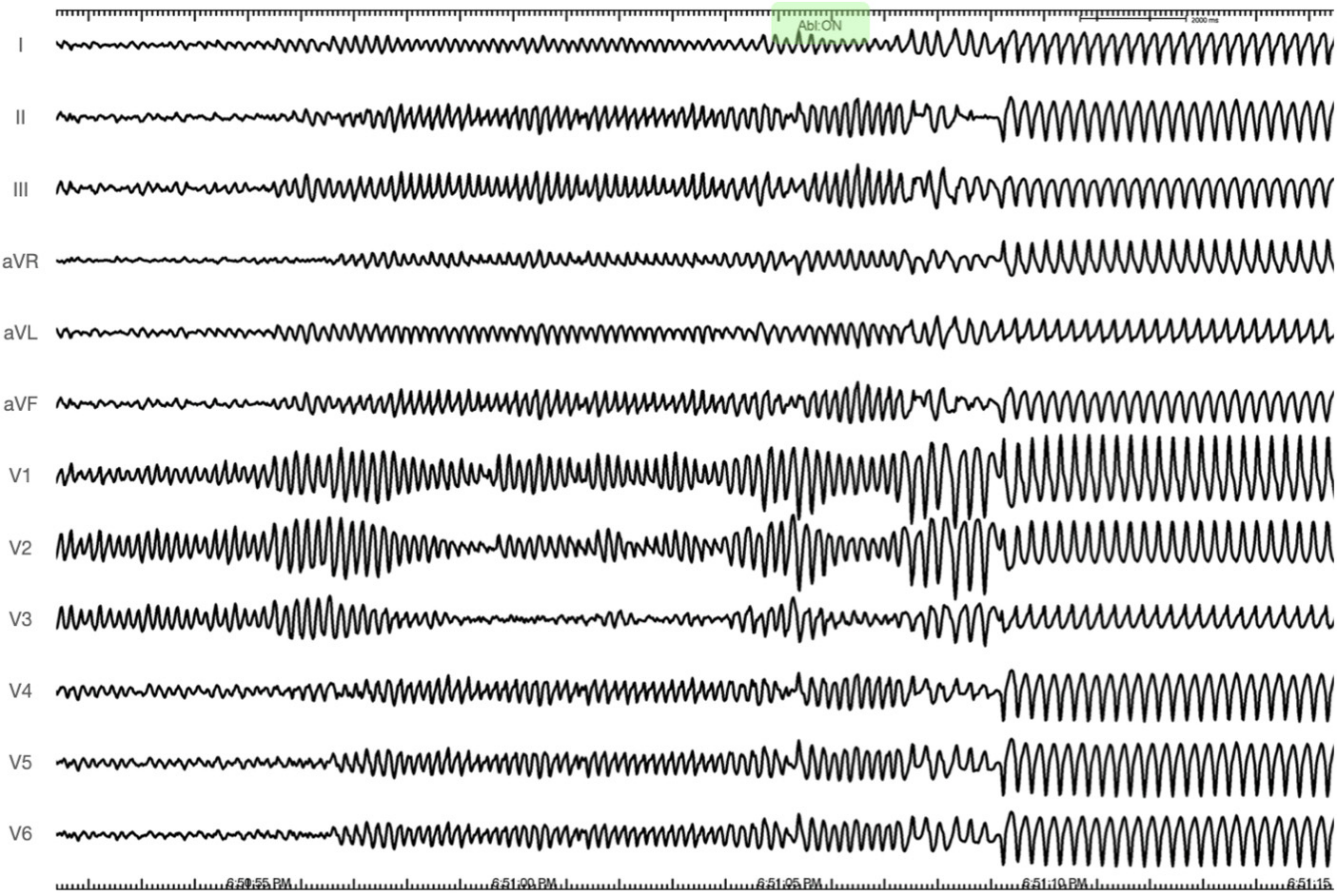
12-Lead Electrocardiogram of Sustained Polymorphic/Ventricular Fibrillation Preceding the Initial Application of Radiofrequency Energy

**Figure 3:**
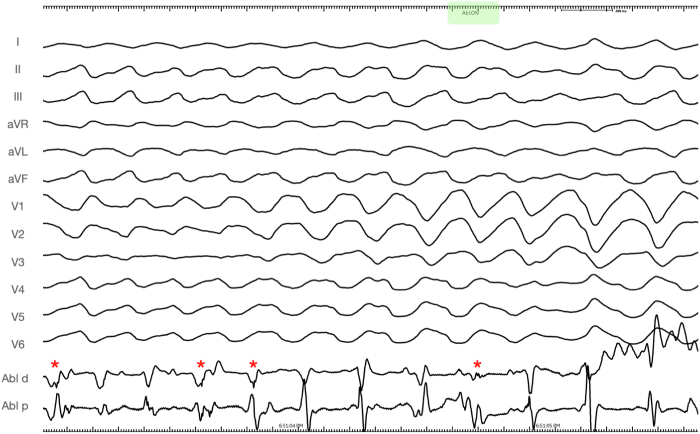
Local Electrogram Recorded at Successful Moderator Band Site Intracardiac electrocardiogram recorded from the tip of the ablation catheter (Abl d) during sustained ventricular fibrillation at 100 mm/s sweep speed. Before radiofrequency delivery, intermittent high-frequency electrogram components, which may be consistent with Purkinje potentials, were recorded **(red asterisks).**

**Figure 4:**
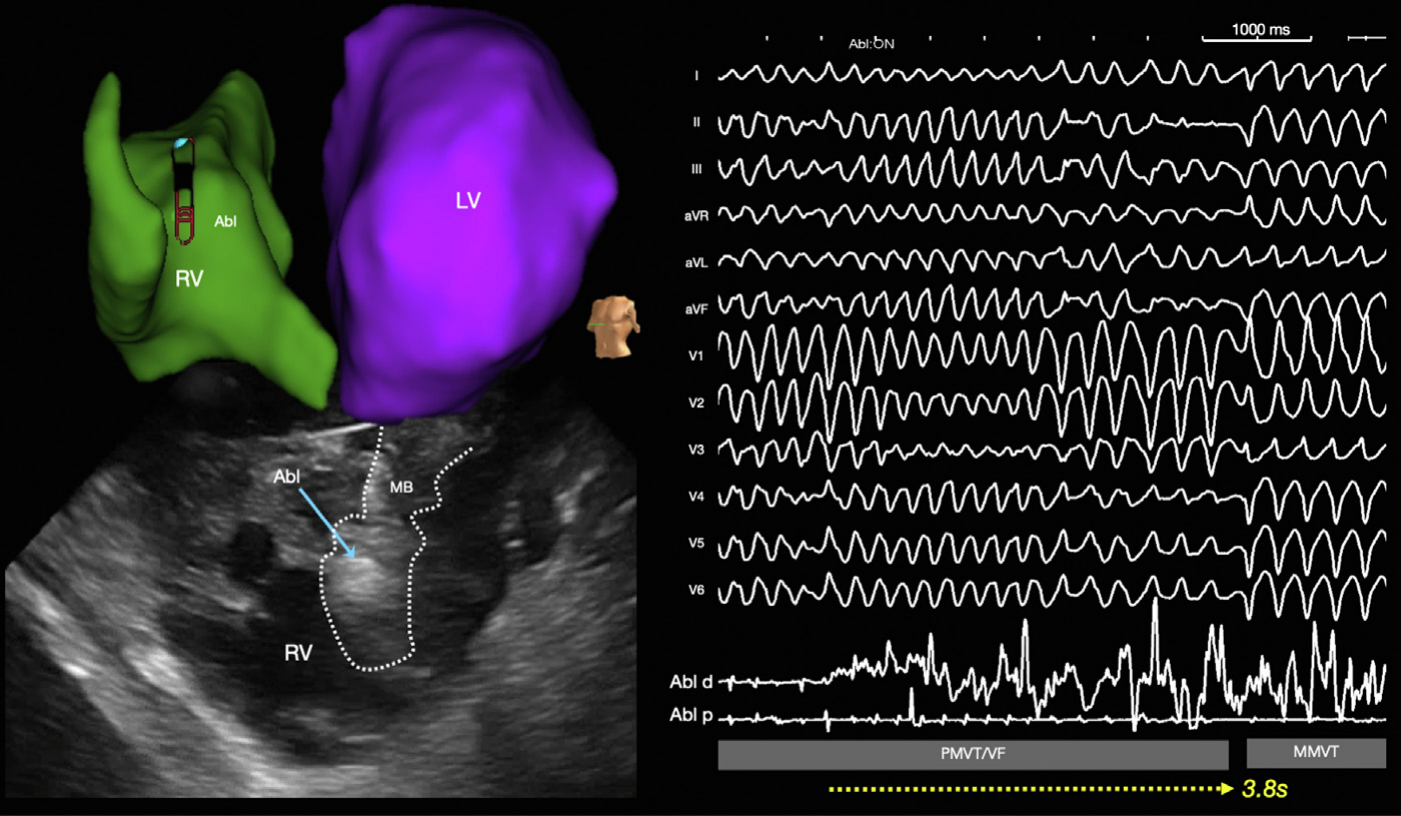
Anatomic Location of Ablation That Resulted in Termination of Ventricular Fibrillation From Moderator Band Electroanatomic map shows ablation catheter (Abl) within the right ventricle (RV). Intracardiac echocardiography confirmed contact with lesion formation (Abl) on the moderator band, which is an intracavitary structure within the RV. During radiofrequency energy delivery, sustained ventricular fibrillation (85 seconds) directly terminated to monomorphic ventricular tachycardia during the delivery of RF ablation in 3.8 seconds of ablation.

In the laboratory, the same stereotyped PVC initiated salvos of VF, requiring defibrillation. Because of suspected moderator band or posterior fascicular trigger, the ablation catheter was first positioned overlying the RV moderator band during an episode of VF. The first radiofrequency (RF) energy was delivered 85 seconds into the sustained VF episode (Figure 2). Intermittent high-frequency electrograms components (red asterisks), that may be consistent with Purkinje potentials were recorded (Figure 3), which resulted in immediate termination of VF directly into monomorphic VT within 3.8 seconds of RF application (Figure 4).

**Figure 5:**
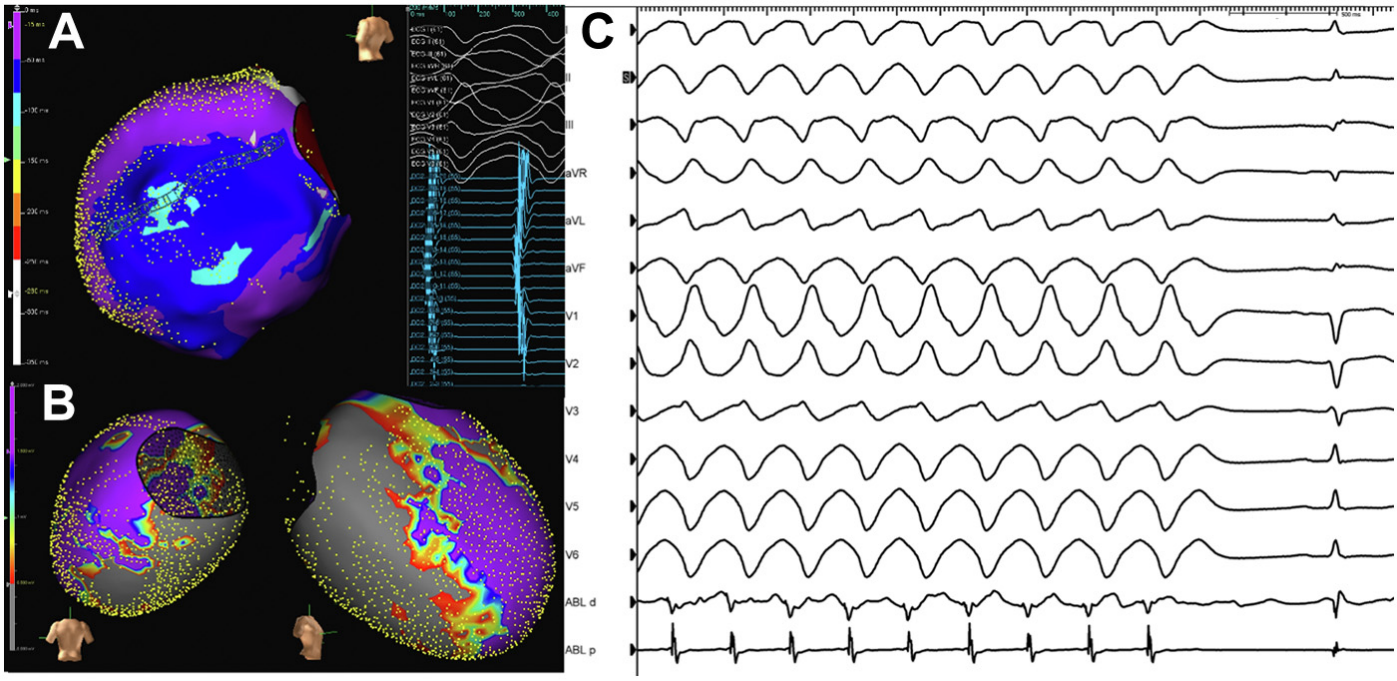
Mapping of Exit Region of Monomorphic Ventricular Tachycardia Within Extensive Inferior Scar Substrate **(A)** The resulting monomorphic ventricular tachycardia was mapped to an exit site lateral to the posteromedial papillary muscle with multielectrode catheter. The activation map and local signals (−65 ms) on the duodecapolar catheter suggest a focal breakout at the site of electrode pair 12 to 13. **(B)** Extensive low-voltage zone involving inferior wall from septum to lateral wall was detected with substrate mapping. **(C)** Termination of the exit region returned the patient to sinus rhythm. The sinus rhythm electrogram is low voltage without Purkinje or late potentials.

The monomorphic VT was then mapped to an endocardial exit site showing focal centrifugal activation lateral to the posteromedial papillary muscle (Figure 5A). Overdrive pacing from the presumed exit site demonstrated concealed fusion and a short postpacing interval—total cycle length of 18 milliseconds. The preablation signal was 65 milliseconds pre-QRS at this location, and the second RF application terminated VT to normal sinus rhythm in 7 seconds (Figures 5A and 5C). The interpretation of these findings on activation and entrainment mapping was a re-entrant mechanism with exit site termination. The sinus rhythm electrogram at the termination site did not exhibit Purkinje or late potentials and was within the low voltage region of extensive inferior scar (<1.5 mV) (Figure 5B). No cardioversion or pacing maneuvers were required to restore sinus rhythm. The stereotypic PVC then again initiated VF which required defibrillation, after which the PVC was no longer observed. Based on pace mapping from the posterior fascicle with similar morphology to the clinical PVC, additional RF ablation was delivered to performed limited linear set with the aim of transecting one portion of the fascicle. No further ventricular arrhythmias were observed.

## Discussion

To the best of our knowledge, this is the first reported observation of direct termination of sustained VF during delivery of RF energy. VF can be subdivided into VF associated with structural heart disease and VF in the setting of no apparent cardiomyopathy, termed *idiopathic VF*. In the present case, the patient presented with a clearly defined substrate of ischemic cardiomyopathy. In the setting of VF or PMVT and ischemic cardiomyopathy, it is critical to first exclude and reverse active ischemia. Another consideration is the initiation of quinidine, specifically in patients with VF in the post-MI setting.^1^ Similar to observations in idiopathic VF, VF after MI is induced by short-coupled extrasystoles arising from the Purkinje network. The transient outward potassium current I_to_ is highly active in these cells, and its inhibition may be the mechanism by which quinidine has a therapeutic effect in these patients.^1^ In the present case, quinidine was initiated and led to longer periods of sinus rhythm between defibrillation but without cessation of VF episodes.

In cases of refractory electrical storm, catheter ablation of VF after MI should be considered. Ablation of idiopathic VF has been well-described and performed by targeting early coupled PVC triggers that usually arise from the Purkinje network.^2^ More recently, catheter ablation of VF storm after MI has been described with a similar approach of targeting PVCs arising from the fascicles at the border region of scar.^3^^,^^4^ In this context, 2 reported cases observations have shown mechanically supported VF that self-terminated in the EP laboratory after RF catheter ablation to sites of Purkinje activation.^5^^,^^6^ However, the present case may be the first in which VF directly and promptly terminated to monomorphic VT during delivery of RF energy to the Purkinje network/moderator band. Although it has been well-known that abnormal Purkinje potentials and resultant PVCs may serve as initiators of VF, this case presents mechanistic evidence that the triggering focus or Purkinje network may in fact be critical to VF perpetuation.

The presented case is unusual, because prior reports of Purkinje-initiated VF typically arise from the border zone of the infarction. Although the moderator band may seemingly be an anatomically remote trigger, RV infarction may have contributed to this unique substrate and clinical presentation. The mechanisms underlying transition of fibrillation into an organized rhythm, akin to termination of atrial fibrillation into atrial flutter, remain incompletely understood. There may be concurrent activation of 2 arrhythmias with unmasking of re-entry upon removal of a trigger, or the final beats of fibrillation may have induced VT. Further studies are needed to replicate these results and elucidate the mechanisms of VF.

## Follow-Up

After catheter ablation, all antiarrhythmic medications were discontinued, and the patient remained free of VT and VF. After an 8-week hospitalization, he expired caused by infectious complications of his prolonged intubation.

## Conclusions

As previous studies have reported, catheter ablation of refractory VF storm can be highly effective in selected refractory cases. However, the literature to date have described ablation of PVC triggers during sinus rhythm as the strategy for preventing recurrent VF. This novel report of direct termination of sustained VF during a single application of radiofrequency energy into MMVT demonstrates that interruption of VF is a mechanistic possibility with ablation. A second radiofrequency application promptly terminated VT to sinus rhythm without the requirement for defibrillation. Anatomic structures that have been established as triggers for VF, such as the moderator band, may also play a mechanistic role in the perpetuation and maintenance of sustained VF.

## Funding Support and Author Disclosures

The authors have reported that they have no relationships relevant to the contents of this paper to disclose.
